# Plasma membrane changes during programmed cell deaths

**DOI:** 10.1038/cr.2017.133

**Published:** 2017-10-27

**Authors:** Yingying Zhang, Xin Chen, Cyril Gueydan, Jiahuai Han

**Affiliations:** 1 State Key Laboratory of Cellular Stress Biology, Innovation Center for Cell Signaling Network, School of Life Sciences, Xiamen University, Xiamen, Fujian 361005, China; 2 Laboratoire de Biologie Moléculaire du Gène, Faculté des Sciences, Université Libre de Bruxelles, 1050 Brussels, Belgium

**Keywords:** programmed cell death, plasma membrane, mechanism, morphology, immunology

## Abstract

Ruptured and intact plasma membranes are classically considered as hallmarks of necrotic and apoptotic cell death, respectively. As such, apoptosis is usually considered a non-inflammatory process while necrosis triggers inflammation. Recent studies on necroptosis and pyroptosis, two types of programmed necrosis, revealed that plasma membrane rupture is mediated by MLKL channels during necroptosis but depends on non-selective gasdermin D (GSDMD) pores during pyroptosis. Importantly, the morphology of dying cells executed by MLKL channels can be distinguished from that executed by GSDMD pores. Interestingly, it was found recently that secondary necrosis of apoptotic cells, a previously believed non-regulated form of cell lysis that occurs after apoptosis, can be programmed and executed by plasma membrane pore formation like that of pyroptosis. In addition, pyroptosis is associated with pyroptotic bodies, which have some similarities to apoptotic bodies. Therefore, different cell death programs induce distinctive reshuffling processes of the plasma membrane. Given the fact that the nature of released intracellular contents plays a crucial role in dying/dead cell-induced immunogenicity, not only membrane rupture or integrity but also the nature of plasma membrane breakdown would determine the fate of a cell as well as its ability to elicit an immune response. In this review, we will discuss recent advances in the field of apoptosis, necroptosis and pyroptosis, with an emphasis on the mechanisms underlying plasma membrane changes observed on dying cells and their implication in cell death-elicited immunogenicity.

## Introduction

Plasma membrane is central for homeostatic maintenance in mammalian cells. It is a direct barrier against extracellular environment; it harbors protein sensors and receptors transducing extracellular signals to elicit cellular responses; it contains transporters and channels involved in the trafficking of inorganic ions and small water-soluble organic molecules; and it participates in nutrient and macromolecule transport processes such as endocytosis and exocytosis. The loss of plasma membrane integrity would therefore undoubtedly put an end to cellular life.

Cell death can result from activation of intrinsic cell death programs or passive disruption of membrane integrity by damaging environmental forces. Since all passively disrupted cells present a ruptured plasma membrane, which is a feature of the necrotic phenotype, necrotic cell death has long been defined as a form of uncontrolled passive cell death^1^. However, it is now clear, that besides apoptosis — the best characterized type of programmed cell death with an intact plasma membrane, other intrinsic programs can lead to necrotic cell death^2^. A very recent study has even shown that plasma membrane rupture in apoptotic cells undergoing secondary necrosis is also intrinsically programmed^3^.

While apoptosis is generally accepted as a non-inflammatory process, the lytic nature of necrosis leads to the release of intracellular DAMPs (damage-associated molecular patterns) and triggers inflammation^4^. However, distinct programs such as necroptosis and pyroptosis or passive 'mechanical' damages will induce different immunogenic effects^5,6^. Furthermore, the idea that apoptosis is a non-inflammatory form of cell death may not be accurate since accumulating experimental data indicate that apoptosis can also be immunogenic due to the emission of particular DAMPs^7^. Secondary necrosis might only be partially responsible for DAMPs release during apoptosis since certain DAMPs are preferentially released by apoptotic rather than necrotic cells^6^. Therefore, plasma membrane changes during apoptosis and programmed necrosis are clearly more complicated than previously expected.

Apoptosis was an early focus in the field of cell death study. The mechanisms of nuclear condensation, DNA fragmentation, caspase activation, and phosphatidylserine flipping have been intensively studied^8^. Although plasma membrane blebbing and apoptotic body formation are morphological characteristics of apoptosis, mechanistic information regarding those processes remains limited. Recent advances in necrosis study have drawn our attention to the plasma membrane. Translocation of channel or pore proteins to the plasma membrane increases plasma membrane permeability and causes plasma membrane rupture in programmed necrosis^9,10,11,12,13,14,15,16,17,18,19,20,21,22^. Plasma membrane pore formation is also responsible for the secondary necrosis of apoptotic cells^3^. Due to the significant progress in the recent study of plasma membrane changes in different cell death programs, this article will first review updated information on necroptosis, pyroptosis, and apoptosis with an emphasis on the comparison of the accompanying plasma membrane changes. We will then review and discuss how these plasma membrane changes in dying cells elicit immune responses.

## Apoptosis

Apoptosis is the most well-studied cell death program, occurring in almost all tissues and being important for normal development and tissue homeostasis. It features morphological changes such as cell shrinkage, chromatin, and cytoplasmic condensation, nuclear fragmentation, breakage of cells and the subsequent formation of membrane-bound apoptotic bodies^23^. While apoptosis can be induced by a variety of physiological or pathological stimuli and conditions, it is mediated by either the extrinsic death receptor pathway or the intrinsic mitochondrial death pathway^24^. The extrinsic pathway involves the recruitment and activation of procaspase-8, and activated caspase-8 then directly activates the effector caspases such as caspase-3 to initiate the execution process. The intrinsic apoptotic pathway is mediated by the cleavage of BID (BH3 interacting domain death agonist), a BCL-2 homology 3 (BH3)-only protein. Truncated BID subsequently translocates to the mitochondria and activates the BCL-2 family members BAX (BCL-2-associated X protein) and BAK (BCL-2 antagonist/killer). Upon activation, BAX and BAK induce mitochondrial outer membrane permeabilization and the release of proapoptotic mitochondrial contents into the cytoplasm, such as cytochrome *c* and other soluble mitochondrial intermembrane space proteins^25^. Released cytochrome *c* promotes oligomerization of APAF-1 (apoptotic peptide activating factor 1), an adaptor protein containing a caspase recruitment domain (CARD). Heptameric APAF-1 recruits procaspase-9 through the CARD-CARD interaction and forms the apoptosome, leading to proximity-induced activation of caspase-9, which in turn cleaves and activates effector caspases^26^. Crosstalk between the extrinsic and intrinsic pathways could occur as both can use the same execution mechanism to elicit cell death. This common execution pathway is initiated by the cleavage of effector caspases, caspase-3/-6/-7 and results in DNA fragmentation, cytoskeletal reorganization, cytoplasmic condensation, and formation of apoptotic bodies^24,27,28^.

### Events occurring at the plasma membrane of apoptotic cells

**Figure 1:**
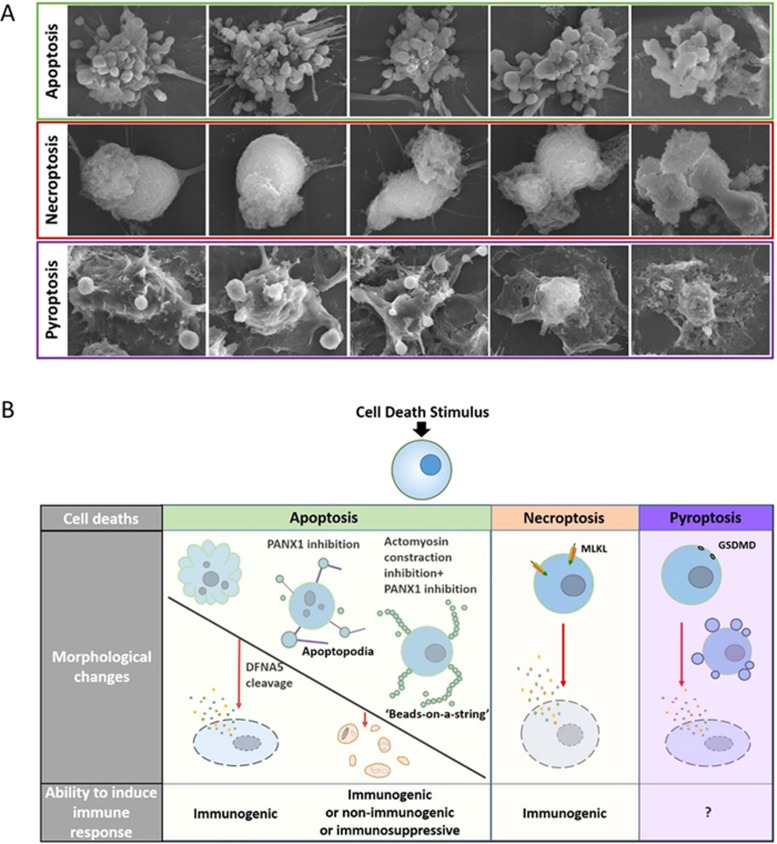
Morphological features of apoptosis, necroptosis, and pyroptosis and their linkages with immunogenicity. **(A)** Dying cells revealed by scanning electron microscopy. In RAW264.7 cells, apoptosis was induced by TNF+Smac mimetics; necroptosis was induced by TNF+Smac mimetics+zVAD; pyroptosis was induced by LPS priming followed by nigericin treatment. **(B)** Membrane blebbing followed by formation of apoptotic bodies is commonly observed in apoptosis. Under certain conditions, such as inhibition of PANX1 by trovafloxacin or further combined inhibition of actomyosin contraction by cytochalasin D or GSK 269962, apoptotic cells exhibit two apoptotic body-related morphological changes called apoptopodia and 'beads-on-a-string' protrusions. These membrane-enveloped fragments can be immunogenic, non-immunogenic, or even immunosuppressive under different experimental settings. However, the regulated secondary necrosis of apoptotic cells mediated by DFNA5 can be highly inflammatory. In necroptosis, MLKL-mediated plasma membrane rupture leads to release of cellular contents and thus immunogenicity. Pyroptosis results from an inflammatory response induced by inflammasome activation, which is frequently observed in professional phagocytes and tightly associated with IL-1β/IL-18 secretion. Whether GSDMD-mediated pyroptosis itself is immunogenic awaits further investigation.

**Figure 2:**
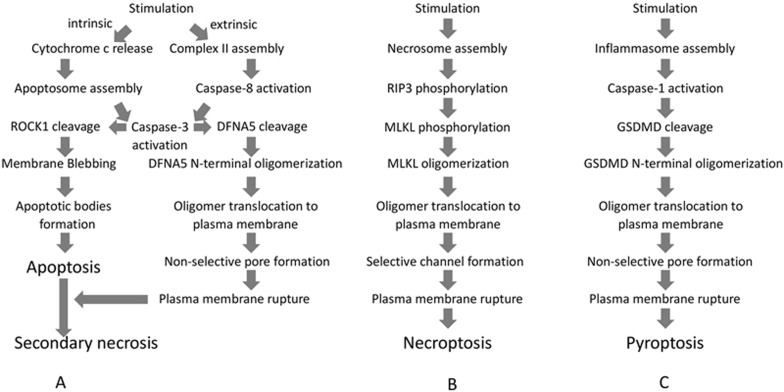
Outlines of the signal transduction pathways leading to plasma membrane changes in apoptosis (including secondary necrosis), necroptosis, and pyroptosis. **(A)** Apoptosis can be initiated by either intrinsic or extrinsic pathway. Caspase-3 activation resulting from either pathway cleaves ROCK1 to promote plasma membrane blebbing, followed by generation of apoptotic bodies. Caspase-3 can also cleave DFNA5 to generate the DFNA5 N-terminal fragment, which forms oligomers and translocates to the plasma membrane, leading to its rupture by the formation of non-selective pores and finally secondary necrosis. **(B)** In the necroptotic pathway, various external death ligands can initiate necrosome assembly. Once in the necrosome, RIP3 is autophosphorylated. Phosphorylated RIP3 recruits and phosphorylates MLKL, leading to MLKL oligomerization and translocation to the plasma membrane. MLKL oligomers execute necroptosis by generating cation channels, causing plasma membrane rupture. **(C)** Pyroptotic stimulation elicits inflammasome formation and subsequent caspase-1 activation. Activated caspase-1 cleaves GSDMD, generating the GSDMD N-terminal fragment, which oligomerizes and translocates to the plasma membrane and causes plasma membrane rupture via non-selective pore formation.

The execution of apoptosis is orchestrated by the proteolytic cleavage of a wide range of cellular substrates by caspases, including cytoskeleton components (such as actin and catenin) and signaling elements^8^. During the final step of apoptotic execution, modifications of the plasma membrane are undoubtedly finely tuned. However, little is known about how dying cells are dismantled. Morphologically, the plasma membrane will first undergo blebbing (formation of circular bulges), a transient stage which rapidly evolves toward bleb separation and generation of apoptotic bodies (Figure 1A). Mechanisms underlying these plasma membrane changes are partly described (Figure 2).

**Membrane blebbing** Caspase-3 has been shown to be necessary for membrane blebbing as caspase-3-deficient cells fail to form membrane blebs^29,30^. Rho-activated serine/threonine kinase ROCK1 is a caspase-3 target^31,32^. Activation of ROCK1 by caspase-3-mediated cleavage is Rho-independent and functions to regulate actin-myosin filament assembly, cell contractility, and membrane blebbing through phosphorylation of the myosin light chain (MLC). ROCK1 plays no role in caspase activation, cytochrome c release, or phosphatidylserine externalization, but ROCK1-dependent membrane blebbing is required for the movement of DNA fragments to blebs and apoptotic bodies. Phosphorylation of MLC by ROCK1 promotes actomyosin contraction with consequential delamination of the plasma membrane from the cortical cytoskeleton membrane, leading to plasma membrane blebbing. Both the loss of the interaction with cytoskeleton and the increase in hydrostatic pressure due to cell shrinkage constitute the physical bases driving membrane blebbing. A few other proteins including p21-activated kinase (PAK) 2 and LIM-kinase 1 (LIMK1) were also reported to be activated by caspase to trigger cytoskeletal reorganization and membrane blebbing^31,33,34,35,36^, but conflicting data exist.

The flipping of phosphatidylserines, a feature of apoptosis, is ROCK1-independent but enriched in apoptotic blebs^31,32^. Although considered as a hallmark of apoptosis and functioning as a 'eat-me' signal to aid apoptotic cell recognition and clearance by phagocytes^37^, phosphatidylserine flipping was reported to be inducible, reversible, and independent of cytochrome *c* release, caspase activation, and DNA fragmentation. It was also suggested that phosphatidylserine flipping may not affect the progression of the apoptotic program^38^. Similarly, blebbing does not affect the development of apoptosis, and is also reported as being reversible^39,40^.

**Apoptotic bodies** Apoptotic bodies are non-uniform subcellular fragments released from apoptotic cells trapping cellular contents such as DNA, fragments of nucleus, and fragmented or intact organelles, the formation of which requires plasma membrane blebbing^31,41^. Apoptotic bodies are ∼1-5 μm in size, and are formed by defined cell types such as T lymphocytes and endothelial cells^37^. However, not all apoptotic cells break up into apoptotic bodies. Indeed, apoptotic cells do not have to be disassembled into sub-particles to be efficiently cleared since evidence shows that in most cases, professional phagocytes tend to phagocytose their targets in their entirety. This is for example the case for macrophages and dendritic cells (DCs) that engulf entire apoptotic thymocytes or neutrophils^42,43^. Non-professional fibroblasts, epithelial, or endothelial cells are also able to phagocytose entirely their dying brethren^44,45^. Thus, the notion that apoptotic bodies form to help clear dead cells still needs to be verified. Cell rounding results from the detachment from the extracellular matrix, a process mediated by caspase-dependent dismantling of cell-matrix focal adhesions and cell-cell adhesion complexes. This event represents the earliest step toward apoptotic body formation. Although the following membrane blebbing is known to be required for apoptotic body formation, the mechanism underlying the formation of these apoptotic bodies is still largely unknown. Caspase-3-mediated activation of gelsolin is believed to mediate in part the morphological changes of apoptosis since the N terminal fragment of gelsolin yielded by caspase3-cleavage causes depolymerization of the actin cytoskeleton in a calcium-independent manner^46^. ROCK1-dependent expansion and retraction of the bleb is involved in debris packaging into the bleb's lumen and finally apoptotic body formation^31,32^.

Pannexin 1 (PANX1) channel is a four-pass transmembrane channel reported to be activated by caspase cleavage and responsible for releasing nucleotide 'find-me' signals, such as ATP, from apoptotic cells to attract phagocytes^47^. In T lymphocytes, impairing PANX1 function by treating cells with trovafloxacin or carbenoxolone, or expressing a PANX1 dominant negative mutant leads to the formation of so-called 'apoptopodia' which are long string-like structures with blebs of different sizes attached to their ends (Figure 1B). Further studies indicate that apoptopodia formation facilitates the separation of blebs to generate apoptotic bodies. Generation of apoptopodia and apoptotic bodies is also observed in LR73 fibroblasts undergoing apoptosis^48^. The dynamic regulation of PANX1 could play a role in apoptotic body formation^48^.

Another study described the formation of 'beads-on-a-string', another type of apoptotic body-related structure^49^. This process is observed during apoptosis of human monocytic-like THP-1 cells subjected to ultraviolet irradiation and during spontaneous apoptosis of primary human CD14^+^ monocytes cultivated under serum-free conditions. Different from the apoptopodia of apoptotic T cells, these 'beads-on-a-string' protrusions extend up to eight times the length of the cell body. The 'beads' on the protrusions could fragment and consequently shear off the 'string' to form apoptotic bodies that are about 1-4 μm in diameter, smaller than those released from T-cell apoptopodia^49^. Further studies uncovered that blockage of PANX1 also promotes 'beads-on-a-string' protrusions and apoptotic body formation in THP-1 monocytes. Strikingly, when T lymphocyte membrane blebbing is prevented by inhibition of actomyosin contraction while at the same time apoptopodia formation is promoted by impairing the function of PANX1, non-beaded apoptopodia can convert to beaded apoptopodia ('beads-on-a-string'), generating smaller apoptotic bodies (Figure 1B). These data suggest that apoptotic bodies and their related structures are dynamically connected and that PANX1 might function not only to regulate ATP release but also to control the formation of apoptotic bodies in different types of cells while the exact mechanism requires further investigation.

**Secondary necrosis** Secondary necrosis is a phenomenon referring to the progressive loss of plasma membrane integrity of apoptotic cells^50^. It is believed to occur in vivo especially in tumor cells from patients undergoing selected chemotherapy and some types of radiation therapy, when apoptotic cells are not efficiently cleared by scavenging cells as also observed in lysosomal disorders, or when cells are infected by apoptosis-inducing pathogens such as vesicular stomatitis virus or encephalomyocarditis virus^3,7,51,52^. Secondary necrosis has long been considered as an unregulated process, but a recent study revealed that caspase-3-dependent cleavage of DFNA5, a GSDMD-like protein, triggers necrosis of apoptotic cells (Figure 2). Cleavage of DFNA5 releases an N-terminal fragment, which assembles into pores in the plasma membrane of apoptotic cells leading to pyroptotic-like necrosis. This pyroptotic-like secondary necrosis is believed to deliver immunogenic effectors such as ATP or HMGB1^3^. Since the expression of DFNA5 is not uniform among tissues and cell types, DFNA5-dependent secondary necrosis could be restricted to particular cell types. At this point, it is unclear if secondary necrosis is programmed in all cell types. If so, it must be sustained by different molecular mechanisms. Concomitant with the identification of DFNA5 cleavage as a determinant of secondary necrosis, another group of investigators also discovered a role of DFNA5 (named GSDME in this context) in chemotherapy drugs-induced caspase-3-dependent cell death^53^. This process was called pyroptosis rather than secondary necrosis since GSDME-mediated cell death is faster than apoptosis. This study further revealed that GSDME is silenced in most cancer cells while expressed in many normal tissues. GSDME-deficient mice are protected from chemotherapy drugs-induced tissue damages and weight loss. These authors proposed that the expression level of GSDME determines whether the cell dies by secondary necrosis (low GSDME) or pyroptosis (high GSDME).

## Necroptosis

Necroptosis, the programmed necrotic cell death initiated by RIP1/RIP3^54^, is known to occur in a variety of pathological conditions^55^. It was first observed in tumor necrosis factor-α (TNF)-treated fibroblast cell line L929^56,57^. TNF is an inducer of both apoptosis and necroptosis^58^. Once it binds to TNF receptor 1, TNF induces receptor trimerization, followed by recruitment of death domain (DD)-containing adaptor proteins TRADD (TNF-receptor associated via DD), TRAF2 (TNF receptor-associated factor 2), and RIP1 (receptor-interacting protein kinase 1) to form the so-called complex I. Subsequently, several components of complex I reshuffle to form a cytosolic complex (complex II), which recruits FADD (Fas-associated via DD) through DD-mediated interaction. Within this complex, FADD recruits procaspase-8 whereas RIP1 recruits RIP3. If RIP3 is absent or present at a low level, caspase-8 is able to undergo auto-activation and the cell undergoes apoptosis. However, in the presence of high concentrations of RIP3, complex II tends to recruit large amount of this protein and turns itself into a so-called necrosome. Procaspase-8 in the necrosome cleaves RIP1 and RIP3, and thus prevents the initiation of necroptosis. This mechanism explains why, in many cases, the occurrence of necroptosis requires the inhibition of caspase-8 activity^59^. Morphologically, necroptosis is marked by organelle swelling and the rupture of plasma membrane (Figure 1A). Mechanistically, the necroptotic program can be initiated either by RIP1-RIP3 hetero-interaction or by RIP3-RIP3 homo-interaction depending on the nature of the stimulus^60,61^. The initial RIP1-RIP3 hetero-complex formation induces the recruitment of additional RIP3 molecules to promote RIP3-RIP3 homo-interaction, which explains the very large size of the necrosome. These RIP3 homo-interactions lead to RIP3 auto-phosphorylation^62^. In return, auto-phosphorylated RIP3 recruits and phosphorylates the mixed-lineage kinase domain-like protein (MLKL)^63,64^. Phosphorylated MLKL subsequently translocates to the plasma membrane, eventually leading to cell lysis (Figure 2)^9,10,11,12^. RIP1 can mediate both apoptotic and necroptotic signaling depending on whether the intracellular RIP3 level is sufficient to convert the signal toward necroptosis^59,65,66^. Since RIP3 expression is cell-type restricted and a high intracellular concentration of this factor is required for initiating the necroptotic program, RIP3 is a key determinant for the initiation of necroptosis^59,67,68^.

### Plasma membrane changes in necroptotic cells

Execution of necroptosis ends up with loss of integrity of the plasma membrane. In the past several years, many details of the molecular mechanism controlling this terminal event were uncovered. A major breakthrough was the identification of MLKL as the executor of plasma membrane rupture. Mechanistic studies reveal that necroptosis is undoubtly an actively controlled cell death process.

**Translocation of MLKL to the plasma membrane** MLKL was identified as a RIP3-interacting protein by mass spectrometry analysis as well as by siRNA screening for proteins involved in the necroptotic pathway^63,64^. MLKL is a pseudokinase containing an N-terminal coiled-coil and a C-terminal kinase-like domain. During the necroptotic process, activated RIP3 recruits and subsequently phosphorylates MLKL (on Thr357/Ser358 in human MLKL and on Ser345/Ser347 in mouse MLKL)^63,69,70^. Because the phosphomimetic mutant of MLKL (hMLKL-T357E/S358D) is predominantly observed in an oligomeric form while the non-phosphorylable mutant (hMLKL-T357A/S358A) remains monomeric and inactive, phosphorylation of MLKL is believed to induce a conformational change that switches MLKL from an inactive to an active state^10^. It is known that phosphorylation of MLKL occurs in the necrosome^63^. Recent data showed that MLKL forms oligomers and departs from the necrosome in its oligomeric form^10,17^. Intermolecular disulfide bonds are present in MLKL oligomers and recent data suggest that MLKL oligomers are octameric structures containing two disulfide-bond-linked tetramers most likely found in a side-by-side position^17^. Accurate formation of MLKL octamers is required for MLKL translocation to the plasma membrane and these octamers span across the plasma membrane^17^.

Besides the formation of oligomeric complexes, the intrinsic property of MLKL to bind lipids is important for the ability of this protein to be addressed to the plasma membrane. Two groups independently found that the N-terminal coiled-coil domain of MLKL directly interacts with cardiolipin (CL) and several types of phosphatidylinositol phosphates (PIPs), such as PI(4)P and PI(4,5)P2^10,12^. Scientists also identified a patch of positively charged amino acids within this region, which is crucial for the lipid binding capacity of MLKL. MLKL does not bind to non-phosphorylated phosphatidylinositol, indicating that the interaction between MLKL and lipids occurs in a charge-dependent manner^10,12^. Although lacking a traditional membrane targeting signal sequence, biochemical fractionation and light microscopy experiments demonstrated that MLKL can be recruited to cellular membranes, especially the plasma membrane^11^. The plasma membrane has the most abundant PI(4)P and PI(4,5)P2 contents. Thus, it is reasonable to speculate that MLKL moves to and preferentially sticks itself into the plasma membrane through its ability to bind PIPs. A recent work provided an interesting model to explain how activated MLKL could interact with the plasma membrane: MLKL oligomerization mediated by the brace region (proximal to the N-terminal helix bundle (NB)) might facilitate plasma membrane targeting; after initial recruitment to the plasma membrane, a conformational change in MLKL rearranges the protein-lipid binding network and subsequently promotes MLKL to function as the effector of plasma membrane permeabilization^71^. Although the three-dimensional structure of MLKL has been solved^13^, a full explanation of how MLKL forms oligomers and how its conformation changes upon plasma membrane interaction requires further structural study on the MLKL oligomers.

**Membrane pores or ion channels** Since MLKL functions at the plasma membrane to execute necroptotic cell death, elucidating the biochemical property of plasma membrane-bound MLKL is essential for understanding how plasma membrane rupture is induced during necroptosis. Full-length MLKL or its N-terminal coiled-coil domain but not its C-terminal kinase-like domain can interact with PIPs- or CL-containing liposomes and mediate the release of Tb^3+^ in the classic liposome leakage assay^10^. Therefore, MLKL N-terminal coiled-coil domain may form membrane-disrupting pores similarly to what has been observed for apoptosis-inducing proteins, such as Bax and Bak. This idea is further supported by the fact that necrosulfonamide (NSA), a chemical targeting the N-terminal domain of human MLKL, can block necroptotic membrane disruption^10^. TRPM7-mediated Ca^2+^ influx was reported to be required for necroptosis execution^9^, but another study suggested that this Ca^2+^ signal is a consequence of Smac mimetic treatment and is not required for necroptosis^72^. Na^+^ influx was detected in necroptosis but not apoptosis by other studies^11^. Using a planar lipid bilayer recording technique, a laboratory showed that MLKL functions as a cation channel since MLKL channels are indistinguishably permeable to Na^+^ and K^+^ but impermeable to Cl−. Further analysis revealed a gradation in MLKL channel permeability to cations as follows: PMg > PNa ≈ PK >> PCa, and PCl^15^. Given the fact that extracellular Na^+^ concentration is much higher than the intracellular Na^+^ level, Na^+^ influx should occur when MLKL channel forms at the plasma membrane. The increase of intracellular osmolality caused by the influx of Na^+^ and other cations might be responsible for the explosion-like plasma membrane rupture observed in necroptotic cell death (Figure 1A)^15,22^. A recent study showed an MLKL-antagonizing function of the ESCRT-III machinery in necroptosis. The ESCRT-III machinery repairs MLKL-mediated plasma membrane damage by shedding damaged plasma membrane as bubbles and thus sustains cell survival^73^. Another study revealed that MLKL actually participates in endosomal trafficking and extracellular vesicle generation^74^. Phosphorylation of MLKL by RIP3 enhances the association of MLKL with endosomes and the interaction with ESCRT may be important for MLKL release within extracellular vesicles^74^.

## Pyroptosis

Pyroptosis is another form of lytic cell death and is defined by its dependence on the inflammasome-mediated activation of caspase-1 and/or -11 in mice (caspase-1 and/or caspase-4/-5 in human). This form of cell death is mostly observed in professional phagocytes, such as macrophages, monocytes, and DCs, but emerging evidence shows that it can be induced in other cell types^75,76^. Inflammasomes are large multiprotein complexes that play critical roles in interleukin (IL)-1β and IL-18 production. Different subsets of inflammasomes are induced by different stimuli. For instance, the NLRP3 inflammasome is thought to be activated by PAMPs, DAMPs, pore-forming toxins, crystals, and UV radiation, while the NLRC4 inflammasome is activated by bacteria expressing flagellin or a type III (T3SS) or IV (T4SS) secretion system. Caspase-1 is a common component of the canonical inflammasomes, while different cytosolic pattern-recognition receptors, including NLRP3, NAIP-NLRC4, NLRP1b, AIM2, or Pyrin, constitute another component. Once assembled, inflammasomes serve as activating platforms for pro-caspase-1. Non-canonical inflammasomes do not recruit caspase-1 and are controlled by caspase-11 (also known as caspase-4 or -5 in human). The non-canonical inflammasomes sense intracellular bacterial lipopolysaccharide and trigger pyroptosis. ASC (also known as PYCARD or TMS-1) is a pyrin- and CARD domain-containing protein that promotes the assembly of certain types of inflammasomes. ASC bridges pro-caspase-1 and pyrin-containing receptors such as NLRP3 and AIM2, while the CARD-containing receptors NLRC4 and NLRP1 can directly interact with pro-caspase-1^77^. Although pyroptosis was identified in 2001^78^, how inflammasome-mediated caspase-1 activation causes a necrosis-like form of cell death was a long-standing question till the identification of GSDMD as the executor of pyroptosis^79,80,81^.

Mechanistically, some similarities can be found between necroptosis and pyroptosis. For example, the execution of both processes is achieved by oligomerization and translocation of pore/channel-forming proteins to the plasma membrane (Figure 2). However, while MLKL, the executor of necroptosis, is activated by phosphorylation, GSDMD, the executor of pyroptosis, is activated by proteolytic cleavage. In the absence of stimulation, full-length GSDMD remains intact with the N-terminal (GSDMD-N) and C-terminal (GSDMD-C) regions interacting with each other. This autoinhibitory conformation is released upon efficient cleavage at a conserved glutamic acid residue (D276 in mouse and D275 in human GSDMD) by caspase-1 or caspase-11, dividing GSDMD into GSDMD-N and GSDMD-C^79,80,81^. The generation of GSDMD-N allows for its oligomerization and only the oligomerized form of GSDMD-N is able to translocate to the plasma membrane and induce cell rupture. The observation that cell death mediated by ectopic expression of GSDMD-N can be inhibited by overexpression of GSDMD-C, reinforces the model of a C-terminal domain-mediated autoinhibition of GSDMD oligomerization^18,19,20,21,22^.

While GSDMD-N kills from within the cell, its released form does not harm neighboring cells. Interestingly, GSDMD-N was reported to have a direct bactericidal effect^19^, consistent with the fact that CL, one of the high-affinity GSDMD-binding lipids, is abundantly present in the inner membrane of bacteria^19^.

### Plasma membrane changes of pyroptotic cells

As a type of lytic cell death, pyroptosis is also characterized by plasma membrane rupture. However, both the mechanism leading to plasma membrane rupture and the morphology of pyroptotic cells are different from those of necroptotic cells (Figure 1A). Unlike necroptosis, plasma membrane rupture during pyroptosis is mediated by the GSDMD pore^18,19,20,21,22^, and is associated with pyroptotic body formation and cell flattening (Figure 1A)^22^. Pyroptosis and the maturation/secretion of IL-1β and IL-18 are two distinct consequences of inflammasome activation, but they share a common molecular mechanism — caspase-1 activation which mediates GSDMD cleavage. Although GSDMD cleavage is required for IL-1β IL-18 secretion, whether the GSDMD pore on plasma membrane is used for the secretion of these cytokines awaits further investigation.

**Pore formation** Although GSDMD shows no significant homology to any known pore-forming toxin, several lines of evidence strongly suggest that GSDMD-N forms functional pores. Recombinant GSDMD-N can permeabilize liposomes of multiple lipid compositions^18,19,20,21^. Using cargoes of different sizes, researchers found that molecules with a diameter of 10 nm or less could pass through GSDMD-N pores. Like MLKL oligomers, GSDMD-N oligomeric complexes bind preferentially to phosphoinositides and CL^18^. Taking advantage of electron and atomic force microscopy, GSDMD-N binding to lipid bilayers could be visualized and analyzed precisely. GSDMD-N typically forms large oligomeric ring-shaped structures minutes after the initiation of full-length GSDMD cleavage^18,19,20,21^. Although some variations were reported by different groups, inner ring diameters of the GSDMD pores were estimated between 10 and 20 nm, consistent with the results obtained from the liposome leakage assay^18,19,20,21^. Different methods have revealed the stoichiometry of GSDMD-N pore to be ∼16 or 24 units^18,21^. As reported for other pore-forming toxins, it is possible that the GSDMD pore contains variable numbers of GSDMD-N monomers. It was assumed that formation of GSDMD pores leads to elevation of the intracellular osmotic pressure, cell swelling, and the eventual plasma membrane rupture, as previously described for MLKL-mediated necroptosis. However, a recent study has shown clear morphological differences between pyroptosis and necroptosis (Figure 1A)^22^. Cell swelling is minimal during pyroptosis and no explosive plasma membrane extension was observed in pyroptotic cells. Instead, pyroptotic cells remain tightly attached to culture slides with flattened cytoplasms. Ion permeability tests revealed that unlike MLKL channels, GSDMD-N pores have no selectivity toward cations or anions^22^. This finding explains why pyroptotic dying cells show little cell swelling but exhibit flattened cytoplasms, as free diffusion of all kinds of ions would not influence the intracellular osmolality^22^.

**Pyroptotic bodies** A novel morphological structure termed pyroptotic bodies has been observed in cells undergoing pyroptosis. Time-lapse electron microscopy examination revealed that pyroptotic bodies were formed before the eventual cell lysis. Compared with apoptotic bodies, pyroptotic bodies have a similar diameter of 1-5 μm^22^. Morphologically, pyroptotic bodies could be categorized as an intermediate class between classic apoptotic bodies and apoptopodia (Figure 1). The discovery of pyroptotic bodies raises several fundamental questions regarding the process controlling the formation and the internal content of these pyroptotic bodies. Released pyroptotic bodies could be engulfed by professional phagocytes, or they could also be the vehicle for information or material transfer among cells. Thus, identification of specific markers or contents of pyroptotic bodies will, with no doubt, help to improve our knowledge on this newly identified cellular structure.

## Immunogenicity of apoptosis, necroptosis, and pyroptosis

It is generally accepted that due to their intact plasma membrane, apoptotic cells are not inflammatory, whereas necrosis is an inflammatory process triggered by the release of cellular contents. However, immunogenicity of apoptosis has been widely reported in the past 20 years^7^. Thus, whether dying cells are immunogenic cannot be simply deduced from the types of cell death.

It is certain that the non-inflammatory nature of apoptosis in organ development is evolutionarily conserved and essential for homeostasis. However, apoptosis triggered by non-natural stimuli can be both pro- and anti-inflammatory. It was shown that uptake of apoptotic neutrophils or eosinophils by macrophages does not induce phagocyte activation and that apoptotic cells even inhibit the production of proinflammatory cytokines by antigen-presenting cells in specific experimental settings^82,83,84,85,86,87,88^. Apoptosis of cancer cells elicited by anticancer therapy could affect the primary tumor environment. Indeed, release from apoptotic cells of cytokines or other factors, such as arachidonic acid and prostaglandin E2 will promote tumor cell growth^89^, and also can lead to accumulation of immune-modulating cells like tumor-associated macrophages^90^. Therefore, apoptotic cancer cells can promote tumor progression^91^. On the other hand, immune-stimulatory or adjuvant-like properties were found for apoptosis induced by certain chemotherapeutics or radiation therapy. This type of apoptosis was named immunogenic apoptosis^92,93,94,95^. The immunogenicity of apoptotic cells relies on the emission of DAMPs, such as calreticulin (CRT), hyaluronate from the degraded stroma, HMGB1 (high-mobility group box 1) from the nucleus, ATP, and heat-shock proteins from the cytosol and formylated peptides and mitochondrial DNA from the mitochondria^96,97^. DAMPs exert their immune-stimulatory effects by stimulating membrane-bound or cytoplasmic pattern-recognition receptors (PRRs, such as toll-like receptors), phagocytic receptors or scavenger receptors (such as LDL receptor-related protein), and purinergic receptors (such as P2RX7/P2RY2). How DAMPs are released by immunogenic apoptotic cells remains largely unknown, although secondary necrosis of apoptotic cells is a very likely mechanism (Figure 1B). Programmed secondary necrosis of apoptotic cells was found to be executed by DFNA5 pores upon activation by apoptotic caspase-3 (Figure 2)^3^. Since DC maturation can be induced by DAMPs together with cancer antigens, promoting secondary necrosis could be an efficient therapeutic approach to induce adaptive immune responses against tumor cells.

Necroptosis is a lytic form of cell death, which releases cellular contents and is therefore considered inflammatory. Numerous intracellular molecules, including DAMPs, become immunogenic after being emitted extracellularly. Accordingly, a recent study has shown that necroptotic cancer cells release DAMPs and promote the production of IFN-γ, maturation of DCs, and cross-priming of CD8^+^ T cells^98^. Another study also demonstrated high vaccination potential of necroptotic cancer cells^99^. Necroptosis is proven to be more immunologically active as compared with apoptosis or 'mechanical' necrosis^98^. Links between necroptosis and inflammation may not only result from the release of DAMPs by lytic cells. A recent work by Cai *et al*.^100^ reported that cleavage of cell surface proteins, including adhesion molecules, by MLKL-activated cell-surface protease of the ADAM (a disintegrin and metalloprotease) family in necroptotic cells may play an important role in initiating the inflammatory responses of necroptosis in addition to the release of cellular contents. Besides, the necroptotic pathway shares some signaling molecules that are also used in regulating the expression of inflammatory genes. For example, RIP1 is involved in necroptosis induced by some stimuli and is also a potent mediator of the activation of the proinflammatory transcription factor NF-κB. A report suggests that NF-κB activation in necroptotic cells is required for necroptotic cell-elicited T-cell cross priming^98^. However, other studies suggested that necroptotic cells themselves are sufficient to trigger T-cell cross priming^6^. Bubbles shed from the plasma membrane of MLKL-activated cells by the ESCRT-III system could mediate danger signaling and activate the immune system^73^. Now, necroptosis is considered as a hallmark of acute and chronic sterile inflammation, such as in the cases of drug- or ischemia-reperfusion-induced tissue injuries and atherosclerosis^101,102^.

Due to its association with IL-1β/IL-18 secretion, activation of pyroptosis is part of the inflammatory response to a number of inflammatory stimuli^76^. Because it is always coupled with the secretion of IL-1β/IL-18, the specific role of pyroptosis in inflammation is not well established. Plasma membrane rupture observed during pyroptosis should induce the release of cellular contents and thus be immunogenic. However, as of today, no experimental data have addressed this issue. Like necroptosis, pyroptosis is also reported to be involved in host defense against severe pathologies caused by intracellular bacteria or viruses^76^. For instance, a specific structure termed pore-induced intracellular trap (PIT) was demonstrated to form from corpses of pyroptotic cells trapping viable bacteria and coordinate innate immune responses to drive neutrophil efferocytosis of the PIT. PITs were also observed during necrosis and necroptosis^103^. Interestingly, recent studies revealed significant differences between these two types of programmed necrosis (necroptosis vs pyroptosis) in terms of membrane rupture^22^. Since plasma membrane plays an important role in extra-/intra-cellular communications, the formation of non-selective pores and pyroptotic bodies during pyroptosis might result in different immune responses than those of necroptosis and apoptosis.

## Concluding remarks

Since apoptosis occurs with preserved plasma membrane integrity, rupture of the plasma membrane was not a major focus in the cell death field until recent progresses in the mechanistic studies of programmed necrosis. The inducible formation of selective ion channels and non-selective pores in necroptosis and pyroptosis, respectively, could be considered a common theme in the execution of necrosis. These channels and pores could initiate changes in plasma membrane permeability and eventually lead to loss of plasma membrane integrity. Loss of the cellular boundary is clearly the most lethal event for cells, which can even apply to apoptosis since secondary necrosis of apoptotic cells was also found to be regulated by the formation of plasma membrane pores. Thus, the molecular mechanisms underlying plasma membrane rupture should be an important topic of further investigations not only for necrosis but also for apoptosis and perhaps other types of cell death. The immunogenicity of dying and dead cells is extremely important in cancer therapy and auto-immune diseases. Release of intracellular contents resulting from plasma membrane permeability change and rupture plays a key role in the induction of immune responses. As current data suggest that different types of cell death are differentially immunogenic, the mechanisms of plasma membrane rupture as well as their different consequences should be a research focus in future studies in the cell death field.
